# Stool and blood metabolomics in the metabolic syndrome: a cross-sectional study

**DOI:** 10.1007/s11306-024-02166-3

**Published:** 2024-09-21

**Authors:** Mariana Ponce-de-Leon, Rui Wang-Sattler, Annette Peters, Wolfgang Rathmann, Harald Grallert, Anna Artati, Cornelia Prehn, Jerzy Adamski, Christa Meisinger, Jakob Linseisen

**Affiliations:** 1 Institute for Medical Informatics, Biometry and Epidemiology, Ludwig-Maximilians-Universität München, Munich, Germany; 2 Epidemiology, Medical Faculty, Universität Augsburg, Augsburg, Germany; 3 Institute of Translational Genomics, Helmholtz Munich, Munich-Neuherberg, Germany; 4 German Center for Diabetes Research (DZD), Partner Neuherberg, Munich-Neuherberg, Germany; 5 Institute of Epidemiology, Helmholtz Munich, Munich-Neuherberg, Germany; 6 Munich Heart Alliance, German Center for Cardiovascular Health (DZHK E.V), Munich, Germany; 7 German Diabetes Center (DDZ), Leibniz Institute for Diabetes Research at Heinrich Heine University Düsseldorf, Düsseldorf, Germany; 8 German Center for Diabetes Research (DZD), Partner Düsseldorf, Munich-Neuherberg, Germany; 9 Research Unit of Molecular Epidemiology, Helmholtz Munich, Munich-Neuherberg, Germany; 10 Metabolomics and Proteomics Core, Helmholtz Munich, Munich-Neuherberg, Germany; 11 Institute of Experimental Genetics, Helmholtz Munich, Munich-Neuherberg, Germany; 12 Department of Biochemistry, Yong Loo Lin School of Medicine, National University of Singapore, Singapore, Singapore; 13 Institute of Biochemistry, Faculty of Medicine, University of Ljubljana, Ljubljana, Slovenia

**Keywords:** Metabolic syndrome, Stool metabolomics, Blood metabolomics, Systemic inflammation

## Abstract

**Introduction/objectives:**

Changes in the stool metabolome have been poorly studied in the metabolic syndrome (MetS). Moreover, few studies have explored the relationship of stool metabolites with circulating metabolites. Here, we investigated the associations between stool and blood metabolites, the MetS and systemic inflammation.

**Methods:**

We analyzed data from 1,370 participants of the KORA FF4 study (Germany). Metabolites were measured by Metabolon, Inc. (untargeted) in stool, and using the AbsoluteIDQ^®^ p180 kit (targeted) in blood. Multiple linear regression models, adjusted for dietary pattern, age, sex, physical activity, smoking status and alcohol intake, were used to estimate the associations of metabolites with the MetS, its components and high-sensitivity C-reactive protein (hsCRP) levels. Partial correlation and Multi-Omics Factor Analysis (MOFA) were used to investigate the relationship between stool and blood metabolites.

**Results:**

The MetS was significantly associated with 170 stool and 82 blood metabolites. The MetS components with the highest number of associations were triglyceride levels (stool) and HDL levels (blood). Additionally, 107 and 27 MetS-associated metabolites (in stool and blood, respectively) showed significant associations with hsCRP levels. We found low partial correlation coefficients between stool and blood metabolites. MOFA did not detect shared variation across the two datasets.

**Conclusions:**

The MetS, particularly dyslipidemia, is associated with multiple stool and blood metabolites that are also associated with systemic inflammation. Further studies are necessary to validate our findings and to characterize metabolic alterations in the MetS. Although our analyses point to weak correlations between stool and blood metabolites, additional studies using integrative approaches are warranted.

**Supplementary Information:**

The online version contains supplementary material available at 10.1007/s11306-024-02166-3.

## Introduction

The metabolic syndrome (MetS) is a cluster of metabolic disorders that confers an increased risk of cardiovascular disease, type 2 diabetes and premature death (Alberti et al., 2009; Guembe et al., 2020; Simmons et al., 2010). Its key components are central obesity, insulin resistance, dyslipidemia and hypertension, although its formal definition has changed since its first description (Ambroselli et al., 2023). The metabolic syndrome constitutes a major public health issue, with the global prevalence varying from 12.5 to 31.4%, according to the definition used (Noubiap et al., 2022).

Research in the last decades has highlighted the role of the gut microbiota in the maintenance of metabolic health and the development of the MetS (Fan & Pedersen, 2020). Although the mechanisms have not been fully elucidated, there is evidence that metabolites produced or modified by the microbiota directly influence host metabolism (Agus et al., 2021). Short-chain fatty acids derived from microbial fiber degradation, for instance, are thought to be protective of dyslipidemia and the MetS (Fechner et al., 2014); while amino metabolism in the gut by microbiota is important for protein and energy homeostasis through multiple mechanisms (Lin et al., 2017; Neis et al., 2015). Stool metabolites, therefore, have been proposed as a functional readout of the complex interplay between host, dietary factors and microbiome (Zierer et al., 2018). Blood metabolites, on the other hand, are also influenced by gut microbiota and are altered during the MetS (Ambroselli et al., 2023; Diener et al., 2022; Shi et al., 2023); their relationship with stool metabolites, however, remains poorly investigated.

In this cross-sectional study we used metabolomics data from 1,370 participants of the KORA FF4 population-based study. We investigated the associations between stool and blood metabolites with the MetS and its components, as well as between metabolites in both biological specimens. In addition, we used high-sensitivity C-reactive protein (hsCRP) levels to assess a possible association between MetS-associated metabolites and systemic inflammation, which has been identified as an important mediator in metabolic diseases (Hotamisligil, 2017). Participants of the KORA FF4 study have been extensively characterized, which allowed us to consider several relevant covariables, such as dietary intake, in our analyses.

## Materials and methods

### Study sample

The KORA (Cooperative Health Research in the Region of Augsburg) FF4 study is the second follow-up study of the fourth KORA health survey (KORA S4) conducted from October 1999 to April 2001. Briefly, KORA S4 included 4,261 participants aged 25–74 years with German citizenship in the city of Augsburg, Germany, and two adjacent counties. Two follow-up examinations were carried out: KORA F4, in which 3,080 participants were examined between October 2006 and May 2008 and KORA FF4, which included 2,279 participants examined between June 2013 and September 2014 (Kowall et al., 2017).

The KORA FF4 study was approved by the Ethics Committee of the Bavarian Chamber of Physicians and all procedures followed the ethical standards of the Declaration of Helsinki. All participants provided written informed consent.

A detailed description of the examination procedures, stool and blood collection is available elsewhere (Breuninger et al., 2022; Mitry et al., 2019a; Yao et al., 2022). Briefly, prior to the study center visit, participants received a collection kit and were instructed to collect stool samples on the day of their visit, if possible, or the evening before. The collection kit included one sterile tube with a DNA stabilizing agent and a second without. Participants were asked to keep the samples refrigerated (4–8 °C) and to complete a questionnaire providing information about the time of collection, description of the sample and problems experienced. Stool samples were received at the study center at the beginning of the visit and immediately deep-frozen at -20 °C, then stored at -80 °C until processing. Samples were excluded if collection instructions were not followed, if the sample was unrefrigerated for more than 3 h or if the participant reported taking antibiotics within the previous 2 months.

Blood samples were collected between 8:00 am and 10:30 am after at least 8 h of overnight fasting into serum gel tubes. After blood withdrawal, the blood samples were kept at 4 °C up to six hours. Until further analyses, serum was stored in liquid nitrogen at − 80 °C in synthetic straws.

Blood and stool metabolomics data was available for 1,370 participants after quality control. Unfasted samples (*n* = 10) were excluded from the final study sample, see Online Resource 1 Fig. S1.

### Untargeted metabolomics in stool samples

Stool samples collected in tubes without added DNA stabilizer were used for metabolite measurement. Details on the preprocessing of stool samples have been previously described (Mitry et al., 2019) and can be found in the Online Resource 1. Stool samples were processed at the Metabolomics and Proteomics core facility at Helmholtz Munich. Metabolites were measured by Metabolon, Inc. (Durham, NC, USA) using LC-MS. Human reference EDTA plasma and stool samples (Seralab, West Sussex, UK) were included across batches for quality control. A total of 1,262 metabolites were measured in the KORA FF4 samples (*n* = 1,413), of which 1,140 were also measured in reference samples. Coefficients of variation (CV) by run day were computed for every metabolite. Metabolites were excluded if: (1) they were not detected in the reference stool samples, and/or (2) they had a median CV greater than 25% (*n* = 248), and/or (3) the CV could not be computed for at least two run days, and/or (4) they had only missing values in the reference samples. Outlier samples (*n* = 2) were defined as metabolite-sample pairs where the distance between the log10-transformed metabolite measurement and the metabolite mean was greater than four times the standard deviation. Outliers, samples with all missing values, and samples for which ≥ 50% of metabolite measurements were low (among the 10% lowest measurements for the given metabolite) were excluded. Metabolite measurements were corrected for the dry weight of the stool sample and scaled to a median equal to one.

The majority of metabolites (72%) had less than 20% of missing values. Fig. S2 (Online Resource 1) shows the distribution of missing values. Metabolites with more than 20% of missing values were excluded from further analysis. The rest of missing values was imputed using k-nearest neighbors with variable selection (Faquih et al., 2020) following the schema in Fig. S3 (Online Resource 1). After excluding unannotated metabolites and xenobiotics, 376 metabolites were kept for further analysis. Summary statistics are presented on Table S1 (Online Resource 2). Imputed values were log2 transformed.

### Targeted metabolomics in blood samples

Metabolite profiling in serum samples was performed for KORA FF4 between February and October 2019 using the AbsoluteIDQ^®^ p180 kit (BIOCRATES Life Sciences AG, Innsbruck, Austria), which allowed the simultaneous quantification of 188 metabolites. Details on sample preparation and assay procedure have been described previously (Haid et al., 2018; Zukunft et al., 2018). Metabolites that met the following quality control criteria were kept for further analysis (*n* = 146): (1) average CV in reference samples lower than 25%; (2) concentrations above the corresponding limit of detection (LOD) in at least 50% of samples; LOD was defined for each plate as three times the median value of water-based samples (phosphate buffered saline) included in each plate; and (3) rate of missing values below 5%. Missing values were randomly imputed by values ranging from 75 to 125% of half of the lowest measured value of the corresponding metabolite in each plate.

To minimize plate effects, metabolites were normalized using a plate normalization factor, calculated by dividing the mean of reference sample values in each plate by the mean of all reference samples in all plates. Presence of multivariate outliers was assessed by calculating the mahalanobis distance with the *mahalanobis_distance* function from the rstatix R package. Six samples were identified as extreme outliers by visual examination of a QQ-plot of squared mahalanobis distances vs. a scaled chisquare distribution and excluded from analysis (Online Resource 1 Fig. S4). Summary statistics of metabolite measurements are presented on Table S2 (Online Resource 2). Metabolite levels were log2 transformed and scaled using the function *scale* of the base R package.

### Definition of metabolic syndrome

The metabolic syndrome was defined according to the International Diabetes Federation 2006 consensus (Alberti et al., 2006), which states that for a person to be defined as having the metabolic syndrome they must have central obesity (waist circumference ≥ 94 in males, ≥ 80 in females) plus any two of the following four factors: raised triglycerides (≥ 150 mg/dl), reduced HDL cholesterol (≤ 40 mg/dl in males and ≤ 50 mg/dl in females), raised blood pressure (BP, systolic ≥ 130 or diastolic ≥ 85 mm Hg, previously diagnosed hypertension or medication use) or raised fasting plasma glucose (≥ 100 mg/dl, previously diagnosed type 2 diabetes or medication use). Missing values in continuous variables related to the components of the MetS were imputed with the mean (Online Resource 2 Table S7).

Measurement of KORA FF4 variables has been described before (Huemer et al., 2020). Waist circumference and blood pressure were measured at the study center by trained examiners. Fasting blood samples were used to determine glucose, high-density lipoprotein (HDL) and triglycerides. HDL was assessed in fresh serum by an enzymatic method (AHDL Flex, Dade Behring). Triglycerides were assessed using the Boehringer GPO-PAP assay. Glucose was measured by an enzymatic, colorimetric method using the GLU assay on a Dimension Vista 1500 instrument (Siemens Healthcare Diagnostics) or GLUC3 assay, on a Cobas c702 instrument (Roche).

High-sensitive C-reactive protein was measured from frozen plasma from fasting blood samples using a high-sensitive latex-enhanced nephelometric assay (BN II Analyzer, Dade-Behring/Siemens).

### Covariables

Trained medical interviewers collected information on physical activity, smoking status and alcohol consumption, as part of a structured health interview. Physical activity was categorized in four levels (< 1 h/week, 1 h/week, 1 h/week regularly, >2 h/week) based on leisure time exercise per week during summer and winter. Smoking status is given as never, former and current smoker. Alcohol intake in grams per day was estimated from self-reported consumption of alcoholic beverages on the previous workday and during the previous weekend.

Usual dietary intake was calculated based on one food frequency questionnaire and up to three 24 h food lists per participants. For details see Mitry et al. (2019a, b). In this study, we used the Alternate Healthy Eating Index (AHEI) 2010 to account for dietary pattern. Details on the calculation of the score are given elsewhere (Wawro et al., 2020). Briefly, a modified AHEI 2010 (excluding trans-fat) was calculated based on usual intake estimates of 10 food components: vegetables, fruits, whole grains, sugar-sweetened beverages and fruit juice, nuts and legumes, red/processed meat, fish, polyunsaturated fatty acids, sodium and alcohol.

### Statistical analysis

Separate multiple linear regression models were fitted for each metabolite level as outcome and the presence or absence of the metabolic syndrome as exposure. To assess the relevance of individual MetS components, the continuous variables related to each component were regressed against all others; the residuals from each regression were then extracted, scaled using the function *scale* of the base R package and used as exposures in the models with only MetS-associated metabolites as outcomes. In total, six continuous variables were used: waist circumference, triglycerides, HDL cholesterol, systolic blood pressure, diastolic blood pressure and fasting plasma glucose. All models were adjusted for the following covariables: dietary pattern, age, sex, physical activity, smoking status and alcohol intake. To explore the relationship between the metabolites and systemic inflammation, we fitted separate multiple linear regression models using hsCRP levels as outcome and each MetS-associated metabolite levels as exposure. The model was adjusted for the presence of the MetS as well as for inflammation-related drug intake (anti-hypertensives, aspirin, lipid-lowering medications, glucose-lowering drugs and pain relievers, see Online Resource 2 Table S9) in addition to the covariables mentioned above. False discovery rate (FDR) adjusted p-values were calculated with the Benjamini-Hochberg procedure using the *p.adjust* function of the stats R package for every set of regression models. FDR adjusted confidence intervals were calculated using the Benjamini and Yekuteli algorithm as implemented in Jung et al. (2011). FDR-adjusted p-values less than 0.05 were considered significant.

Over-representation analysis was conducted using the online Metaboanalyst tool with metabolite sets based on KEGG human metabolic pathways (80 sets). Metaboanalyst allows for two feature types to be analyzed: lipids and other metabolites; a separate analysis was conducted for each feature type. Choline was included in the metabolite pathway analysis even if it is listed as a lipid in the annotation provided by Metabolon. The following stool metabolites were not found in the Metaboanalyst pathway libraries and metabolite sets: propionylglutamine, N6-formyllysine, N6-carboxyethyllysine, N-trimethyl 5-aminovalerate, 2-hydroxybutyrate/2-hydroxyisobutyrate, Trimethylamine N-oxide, sphingadienine, hexadecasphingosine (d16:1), sphingosine, N-oleoyl-sphingosine (d18:1/18:1), hexadecatrienoate (16:3n3), CAR 24:0, glycosyl-N-(2-hydroxynervonoyl)-sphingosine (d18:1/24:1(2OH)), eicosanoylsphingosine (d20:1), glycerophosphoserine, myo-inositol. FDR-adjusted p-values less than 0.05 were considered significant.

Spearman’s partial correlation coefficients between stool and blood metabolites (untransformed) were calculated using the *pcor* function from the ppcor R package. Correlations were considered significant when p value < 0.05. The network was built using Cytoscape version 3.9.1, restricted to coefficients with absolute values larger than 0.2 and only showing correlations across groups (blood, stool). MOFA model was built using the MOFA2 R package (see Online Resource 1 for model parameters). All analyses were conducted in R version 4.3.0.

## Results

The characteristics of the study sample are given in Table 1. The KORA FF4 study is the second follow-up of the KORA S4 cohort and consists mainly of middle-aged and old adults. The study sample is balanced with respect to sex and has a median age of 59 years. The MetS, as defined in this study, is present in 33% (*n* = 457) of the study participants, which are mostly male (*n* = 286). The most common combination of MetS components is central obesity with raised fasting glucose and raised blood pressure (*n* = 201), followed by participants who in addition have raised triglycerides (*n* = 83) (Online Resource 1 Fig. S1).

**Table 1:** Characteristics of study participants

	No MetS (*n* = 913)	MetS (*n* = 457)	Overall (*n* = 1370)
**Age (years)**	55.0 [47.0, 66.0]	65.0 [57.0, 74.0]	59.0 [49.0, 69.0]
**Sex (female)**	505 (55.3%)	171 (37.4%)	676 (49.3%)
**Waist to hip ratio**	0.879 [0.815, 0.934]	0.966 [0.914, 1.02]	0.909 [0.844, 0.970]
**Fat mass index**	17.5 [16.1, 19.4]	19.9 [18.3, 21.4]	18.4 [16.6, 20.2]
Missing	13 (1.4%)	9 (2.0%)	22 (1.6%)
**Hypertension**	194 (21.2%)	326 (71.3%)	520 (38.0%)
Missing	3 (0.3%)	2 (0.4%)	5 (0.4%)
**Heart attack**	14 (1.5%)	36 (7.9%)	50 (3.6%)
Missing	2 (0.2%)	2 (0.4%)	4 (0.3%)
**Stroke**	17 (1.9%)	14 (3.1%)	31 (2.3%)
Missing	1 (0.1%)	1 (0.2%)	2 (0.1%)
**Physical activity**			
< 1 h/week	208 (22.8%)	155 (33.9%)	363 (26.5%)
1 h/week	119 (13.0%)	82 (17.9%)	201 (14.7%)
1 h/week regularly	295 (32.3%)	142 (31.1%)	437 (31.9%)
> 2 h/week	291 (31.9%)	78 (17.1%)	369 (26.9%)
**Smoking**			
Smoker	155 (17.0%)	60 (13.1%)	215 (15.7%)
Ex-smoker	304 (33.3%)	187 (40.9%)	491 (35.8%)
Non-smoker	454 (49.7%)	210 (46.0%)	664 (48.5%)
**Alcohol (g/day)**	7.00 [0.210, 20.9]	5.71 [0, 24.4]	5.93 [0, 22.9]
**Fasting glucose (mmol/l)**	5.22 [4.94, 5.50]	6.00 [5.72, 6.55]	5.44 [5.11, 5.88]
**Systolic blood pressure (mmHg)**	114 [106, 123]	129 [117, 140]	118 [108, 130]
**Diastolic blood pressure (mmHg)**	71.5 [66.0, 77.5]	76.0 [69.0, 82.5]	72.5 [66.6, 79.0]
**AHEI**	45.6 [39.0, 51.6]	43.3 [37.3, 48.7]	44.8 [38.6, 50.7]
Missing	249 (27.3%)	141 (30.9%)	390 (28.5%)
**HbA1c (%)**	5.35 [5.08, 5.54]	5.63 [5.44, 6.08]	5.44 [5.17, 5.72]
Missing	5 (0.5%)	1 (0.2%)	6 (0.4%)
**HDL (mg/dl)**	68.0 [57.0, 81.9]	53.0 [45.0, 65.1]	63.0 [51.7, 77.0]
**LDL (mg/dl)**	132 [109, 155]	135 [110, 162]	133 [109, 157]
Missing	1 (0.1%)	0 (0%)	1 (0.1%)
**Triglycerides (mg/dl)**	90.0 [69.0, 117]	148 [104, 199]	104 [76.7, 143]
**Cholesterol (mg/dl)**	215 [191, 239]	213 [186, 244]	214 [189, 240]
Missing	1 (0.1%)	0 (0%)	1 (0.1%)
**GGT (U/L)**	21.0 [15.0, 32.0]	33.0 [22.7, 54.2]	24.8 [16.4, 38.9]
**GPT (U/L)**	21.0 [16.0, 28.0]	27.0 [21.0, 39.0]	23.0 [18.0, 31.0]
**Uric acid (mg/dl)**	5.16 [4.32, 6.08]	6.39 [5.33, 7.36]	5.50 [4.57, 6.62]
Missing	1 (0.1%)	0 (0%)	1 (0.1%)
**Creatinine (mg/dl)**	0.860 [0.750, 0.970]	0.930 [0.810, 1.07]	0.880 [0.770, 1.01]
Missing	1 (0.1%)	0 (0%)	1 (0.1%)
**High-sensitive C-reactive protein (mg/L)**	0.870 [0.440, 1.92]	1.67 [0.908, 3.60]	1.12 [0.560, 2.42]
Missing	0 (0%)	1 (0.2%)	1 (0.1%)
**Lipid-lowering medication intake**	102 (11.2%)	119 (26.0%)	221 (16.1%)
Missing	3 (0.3%)	1 (0.2%)	4 (0.3%)
**Diabetes medication intake**	12 (1.3%)	97 (21.2%)	109 (8.0%)
Missing	3 (0.3%)	1 (0.2%)	4 (0.3%)
**Aspirin intake**	68 (7.4%)	85 (18.6%)	153 (11.2%)
Missing	3 (0.3%)	1 (0.2%)	4 (0.3%)
**Pain relievers intake**	23 (2.5%)	16 (3.5%)	39 (2.8%)
Missing	3 (0.3%)	1 (0.2%)	4 (0.3%)
**Anti-hypertensives intake**	3 (0.3%)	12 (2.6%)	15 (1.1%)
Missing	3 (0.3%)	1 (0.2%)	4 (0.3%)

Data is given as median [25^th^ percentile, 75^th^ percentile] or count (percentage). AHEI, Alternate Healthy Eating Index; HbA1c, hemoglobin A1c; HDL, High-density lipoprotein; LDL, Low-density lipoprotein; GGT, Gamma-glutamyl transferase; GPT, Glutamic-pyruvic transaminase

**Fig. 1:**
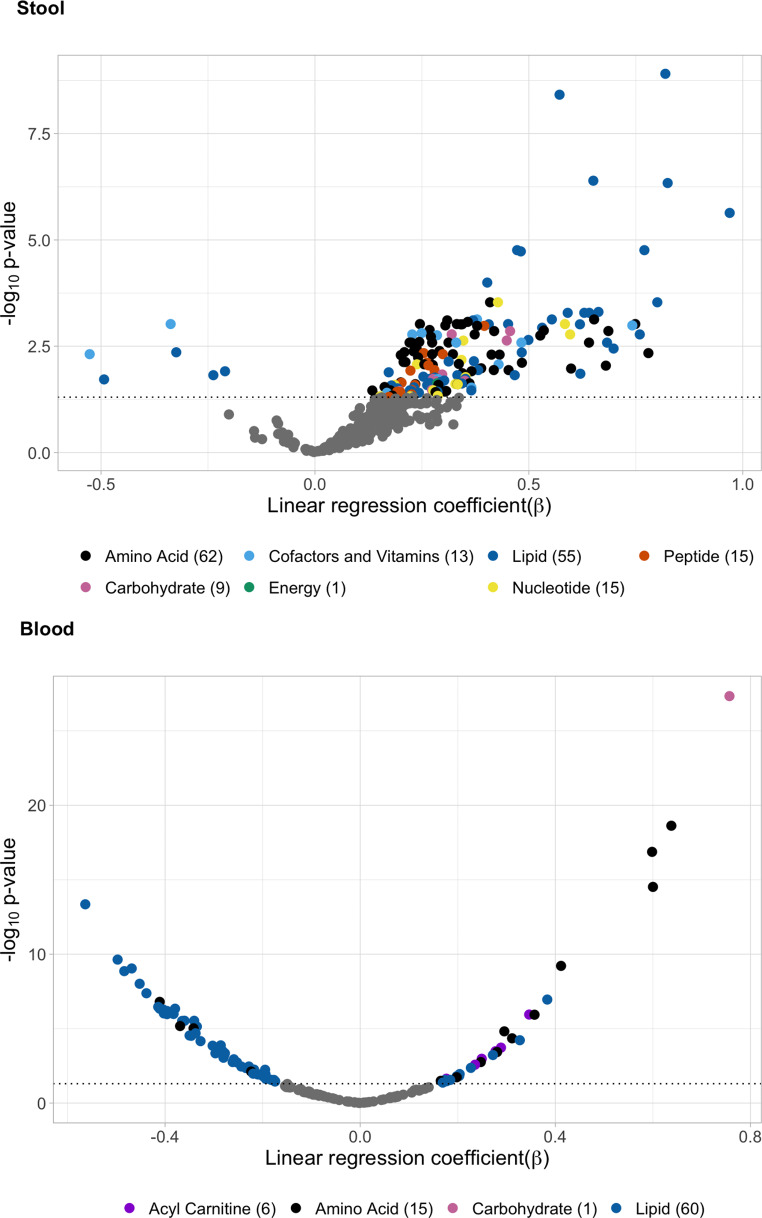
Stool and blood metabolites and the metabolic syndrome. Volcano plots showing results from multiple linear regression analysis for stool and blood metabolites. Dashed line indicates statistical significance threshold adjusted for false discovery rate (FDR). Metabolites with a significant association are colored based on biochemical class

We found 170 stool metabolites associated with the MetS after linear regression analyses (Fig. 1, Online Resource 2 Table S3). Most of the metabolites were amino acids (*n* = 62), followed by lipids (*n* = 55), and showed a positive association with the MetS (*n* = 164). Pathway enrichment analysis showed significant enrichment in 14 metabolic pathways, 9 of which belong to amino acid metabolism and metabolism of other amino acids (Online Resource 2 Table S8). Analysis of targeted metabolomics in blood showed significant associations of the MetS with 82 metabolites, 60 of which were lipids, mostly phosphatidylcholines. The majority of the associations were negative (*n* = 53) (Fig. 1, Online Resource 2 Table S4).

**Fig. 2:**
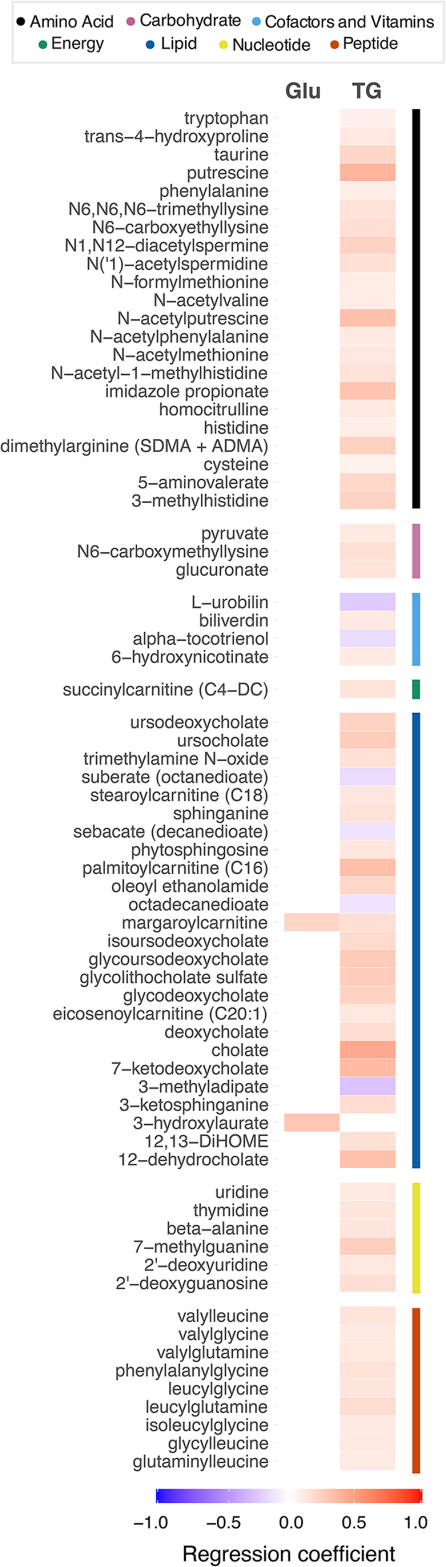
Components of the metabolic syndrome and stool metabolites. Heatmap showing significant associations between individual MetS components and stool metabolites. Associations were considered significant when FDR-adjusted P values < 0.05. Regression coefficients, from multiple regression analyses, are represented by colors ranging from red (positive) to blue (negative). Metabolites are grouped by biochemical classes provided by Metabolon. Glu, fasting glucose; TG, triglycerides

**Fig. 3:**
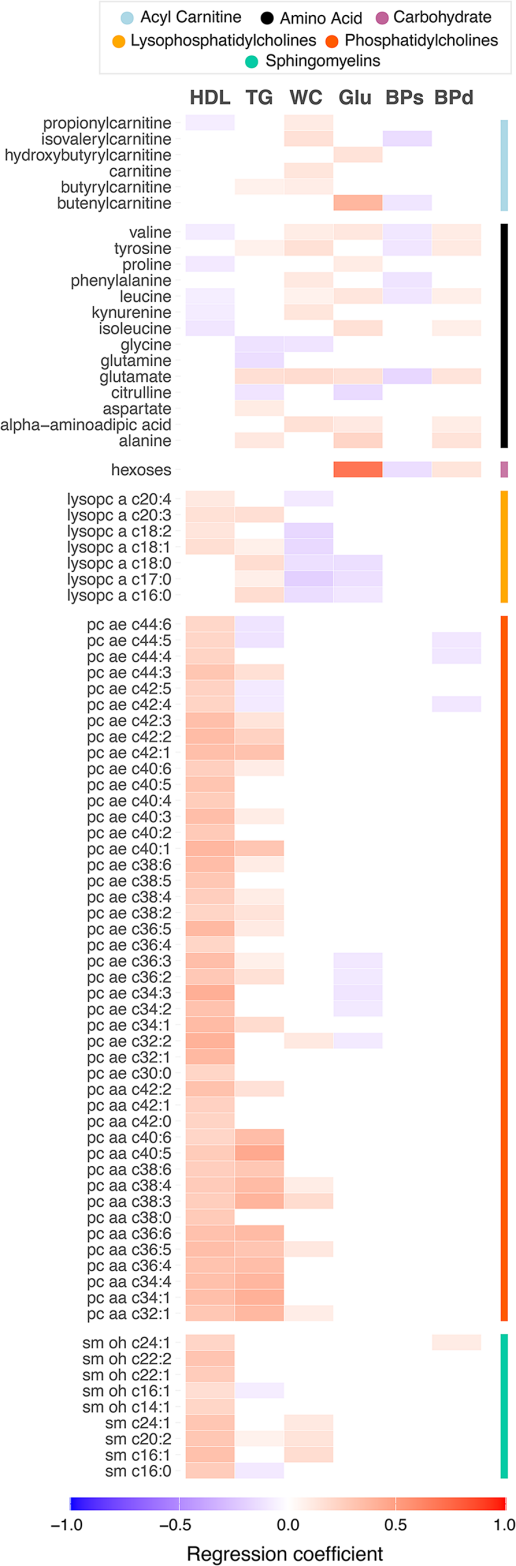
Components of the metabolic syndrome and blood metabolites. Heatmap showing significant associations between individual MetS components and blood metabolites. Associations were considered significant when FDR-adjusted P values < 0.05. Regression coefficients, from multiple regression analyses, are represented by colors ranging from red (positive) to blue (negative). Metabolites are grouped by biochemical classes provided by Biocrates. HDL, high-density lipoprotein cholesterol; TG, triglycerides; WC, waist circumference; Glu, fasting glucose; BPs, systolic blood pressure; BPd, diastolic blood pressure

In order to investigate which components of the metabolic syndrome were relevant, we carried out regression analyses using the continuous variables related to each component. Triglyceride levels were associated with the largest number of MetS-associated metabolites in stool, across all classes of metabolites. Blood pressure, HDL cholesterol and waist circumference did not show any significant associations with MetS-associated stool metabolites (Fig. 2, Online Resource 2 Table S5). HDL cholesterol was associated with the majority of MetS-associated blood metabolites, followed by triglyceride levels. All variables showed at least one significant association with blood metabolites (Fig. 3, Online Resource 2 Table S6).

**Fig. 4:**
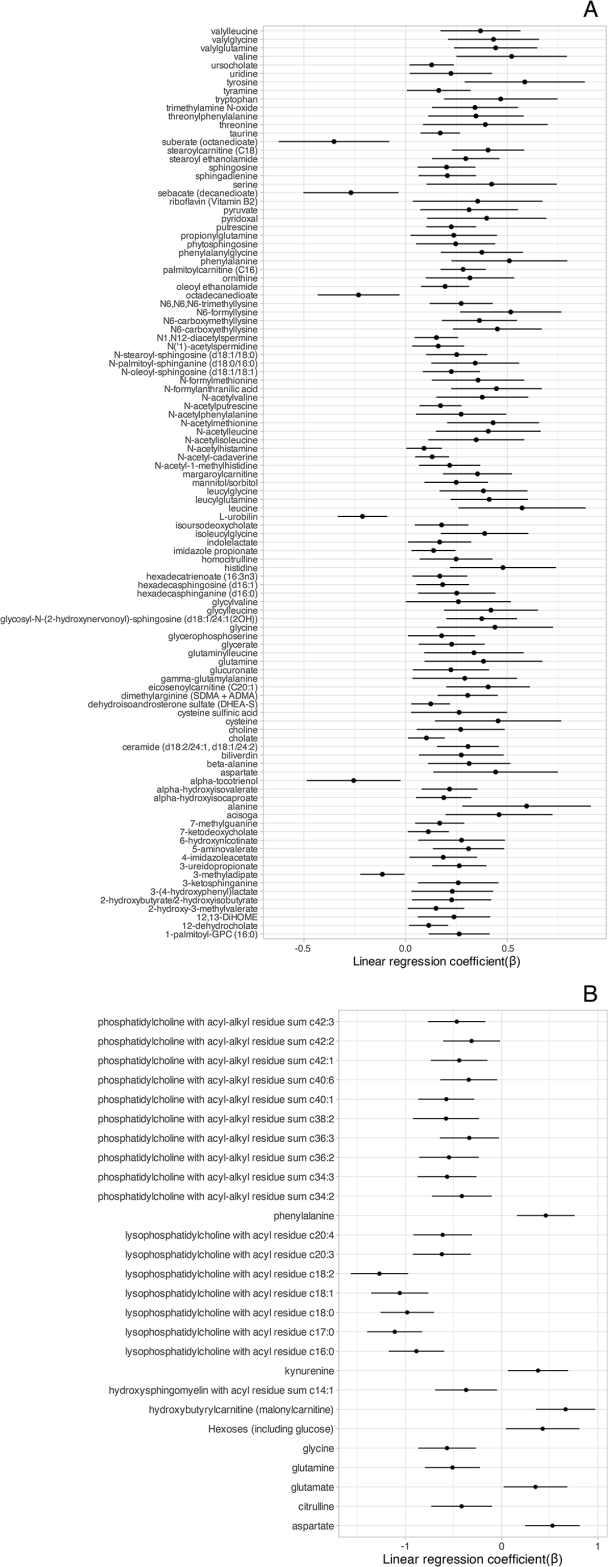
MetS-associated metabolites and systemic inflammation. Results of linear regression analyses investigating the association between MetS-associated stool (**A**) and blood (**B**) metabolites and systemic inflammation. Only significant linear regression coefficients are shown with 95% FDR-adjusted confidence intervals

We tested the hypothesis that MetS-associated metabolites could be related to systemic inflammation using hsCRP levels as biomarker. MetS-associated metabolites showed significant associations with hsCRP levels (107 in stool and 27 in blood), independent of the metabolic syndrome (Fig. 4). Finally, we investigated correlations between stool and blood metabolites, 32 of which overlapped. Partial correlation analyses showed multiple significant correlations; however, only two pairs of metabolites had coefficients larger than 0.20. Trans-4-hydroxyproline (stool vs. blood) was the only overlapping metabolite with a correlation coefficient larger than 0.2. This metabolite was associated with the MetS in stool, but not in blood. The pair L-kynurenine (stool)-creatinine (blood) had the strongest correlation of 0.3 (Online Resource 1 Fig. S5). In addition, we integrated both data sets using a factor analysis model (Argelaguet et al., 2018). This data integration method allows for the identification of variation across two or more data “modalities”. Downstream analysis of the model showed no covariation between blood and stool metabolites (Online Resource 1 Fig. S6).

## Discussion

In this study we used metabolomics data from a population-based study to explore the relationship between stool and blood metabolites with the MetS, its components and systemic inflammation. Multiple biochemical classes of stool and blood metabolites were associated with the MetS, particularly with dyslipidemia. Many of these metabolites were also associated with systemic inflammation. Stool and blood metabolites did not show strong covariation.

### Stool metabolomics

#### Amino acid metabolism

Multiple studies have reported altered blood amino acid levels in the metabolic syndrome (Cheng et al., 2021; Ntzouvani et al., 2017; Yamakado et al., 2015). There was an enrichment in several amino acid metabolism pathways among MetS-associated stool metabolites in our study. Some stool metabolites belonging to these pathways, such as polyamines, have been linked to the maintenance of the intestinal epithelial integrity and are elevated in gastrointestinal diseases (Le Gall et al., 2011; Lee et al., 2020; Rao et al., 2020). Information about specific stool amino acid alterations in the metabolic syndrome, however, is lacking.

#### Lipids

Sphingolipids, including ceramides, were the most common subclass of MetS-associated lipids; they showed a positive association and were also associated with systemic inflammation. In addition to being one of the main components of cell membranes, sphingolipids are bioactive lipids with a wide structural and functional diversity. Alterations in their metabolism and their circulating levels have been observed in metabolic disorders, including obesity and type 2 diabetes (Cirulli et al., 2019; Gerl et al., 2019; Khan et al., 2020; Mir et al., 2022). In addition, some sphingolipids, such as ceramide and sphingosine-1-phosphate, have important roles in immunity and inflammation (MacEyka & Spiegel, 2014).

Sphingolipid abundance in stool samples in the context of the metabolic syndrome has been poorly studied. Coleman et al. (Coleman et al., 2022) compared stool lipids of individuals with (*n* = 8) and without (*n* = 10) the MetS and reported differences in the level of three sphingolipids. The set of 1140 metabolites measured in our study (of which 326 were lipids) did not include any of the 20 lipids with the highest fold change reported by the authors, which prevents any comparisons. Differences in extraction and separation methods could explain the fact that these lipids were not measured in our study.

The second most abundant subclass of lipids positively associated with the MetS, particularly with triglycerides, was bile acids. Primary bile acids, cholic acid and chenodeoxycholic acid, are produced in the liver and secreted to the intestine, where they participate in the emulsification and solubilization of lipids. After this, they enter an enterohepatic circulation cycle in which up to 95% of secreted bile acids return to the liver (McGlone & Bloom, 2019). Bile acids that remain in the intestine are deconjugated and transformed into secondary bile acids. A proportion of secondary bile acids is reabsorbed and returns to the liver, the rest is excreted in the feces. The amount of bile acids in the liver and intestines depends on many factors, including diet, synthesis rate, transport, reabsorption and transformation by microbiota (Di Ciaula et al., 2017).

Previous research in our group identified positive associations between bile acids in the stool and microbial subgroups that have been associated with the risk of metabolic disease (Breuninger et al., 2022). However, the relationship between microbiota and bile acid composition and the effect they have on each other is a topic of ongoing research (Cai et al., 2022; Staley et al., 2017; Wahlström et al., 2016).

Bile acids also act as signaling molecules and are recognized as metabolic and immune regulators. It has been suggested that alterations in bile acid composition are involved in intestinal inflammation, particularly in inflammatory bowel disease (Calzadilla et al., 2022). However, most of the evidence comes from in vitro or animal studies and their role is not yet clear. Their inflammatory or anti-inflammatory properties seem to vary depending on the specific bile acid (Calzadilla et al., 2022). In our study, levels of the primary bile acid, cholate, and the secondary bile acids, ursocholate, isoursodeoxycholate, 7-deoxycholate and 12-dehydrocholate in stool were positively associated with systemic inflammation.

### Blood metabolomics

Alterations in the concentration of multiple metabolites, in particular amino acids and lipids, have been described in the MetS before. Negative associations with lipids such as some phosphatidylcholines and sphingomyelins have been reported in KORA (Shi et al., 2023) and other studies (Mir et al., 2022; Surowiec et al., 2019). Our results are generally in line with previous findings pointing to reduced levels of some, but not all, circulating lipids. In our study, we found a preponderance of negative associations with phosphatidylcholines. Discrepancies in the individual lipids identified could be explained by differences in the technology used to measure the metabolites, the definition of the MetS used as well as in the covariables and methods used on the analyses.

A prior study using untargeted metabolomics (Metabolon Inc.) compared individuals with obesity alone (*n* = 39) with individuals with the MetS (*n* = 18) and identified enriched metabolic pathways including the arginine and proline metabolism pathway (Mir et al., 2022). In our study, blood metabolites were measured using a targeted approach, which prevented us from confirming this finding.

We found multiple significant correlations between stool and blood metabolites. However, only two pairs of metabolites showed partial correlation coefficients larger than 0.2. Studies that have examined correlations between stool and plasma metabolites are scarce overall and have mainly been conducted in patient cohorts with small samples (Galié et al., 2021; Xu et al., 2023). The largest and, to our knowledge, the only study to do so in a population-based cohort (Deng et al., 2023) (*n* = 1,007) had similar findings to ours, pointing to low correlations between fecal and stool metabolites. It must be noted that blood metabolites were measured in a targeted fashion in our study and account for a small proportion of the currently quantifiable metabolites, thus studies using untargeted blood metabolomics are warranted.

#### Strengths and limitations

Our study was carried out using data from a population-based sample that comprised well characterized participants. This allowed us to take into consideration multiple covariables in our analyses. Diet, for instance, is an important determinant of both the stool and the blood metabolome and has not been considered in many previous studies. Here, we used dietary patterns based on habitual dietary intake estimates (derived from repeated 24-h food lists and one food frequency questionnaire). In addition, drug intake was accounted for when estimating the effect of metabolite levels on systemic inflammation. Finally, in contrast with previous studies, we used the residual method to assess the association of the continuous variables related to individual components of the MetS, independently of all the others, with stool and blood metabolites.

Our study has several limitations. Both stool and blood metabolite concentrations can be altered during prolonged storage (De Spiegeleer et al., 2020; Haid et al., 2018) and although this is particularly critical for longitudinal studies, we cannot rule out potential bias. Blood samples were kept at 4 °C for up to 6 h, which can affect metabolite stability (Fomenko et al., 2022; Gegner et al., 2022). The stool metabolome is strongly influenced by gut microbial composition and moderately affected by host genetics (Zierer et al., 2018); these factors were not accounted for in this study. Furthermore, the gut microbiota is influenced by ethnicity and demographic factors (Gupta et al., 2017), which may limit the generalizability of our findings. The use of dietary patterns as a proxy for diet quality has its own limitations, which have been discussed before (Mitry et al., 2019b; Wawro et al., 2020). Stool xenobiotics were excluded from our analyses due to a high percentage of missing values and a lack of consensus on imputation procedures. Pathway enrichment analysis is sensitive to several factors such as metabolite misidentification and pathway database choice (Wieder et al., 2021), thus we cannot rule out the presence of false positive results. Lastly, a causal relationship between different metabolites, the MetS, and its components cannot be established due to the cross-sectional design of the study.

In summary, we identified multiple metabolites in serum and stool associated with the MetS, particularly with dyslipidemia. Moreover, alterations in blood and stool metabolites are associated with systemic inflammation. Further studies using integrative approaches are needed to elucidate the relationship between stool and blood metabolites and their specific involvement and clinical significance in the MetS.

## Electronic supplementary material

Below is the link to the electronic supplementary material.

Supplementary Material 1

Supplementary Material 2

## Data Availability

KORA data and biosamples are available upon request by means of a project agreement subject to approval by the KORA Board. Via the KORA.PASST tool, scientists can view and download data dictionaries of the different KORA surveys and submit a proposal.
