# Supplementary material for: Stool and blood metabolomics in the metabolic syndrome: a cross-sectional study
Source: Metabolomics. 2024 Sep 21;20(5):105. doi: 10.1007/s11306-024-02166-3 (PMC11416374; doi:10.1007/s11306-024-02166-3)

Supplementary information for

Stool and blood metabolomics in the metabolic syndrome: a cross-sectional study

Mariana Ponce-de-Leon[1,2#], Rui Wang-Sattler[3,4], Annette Peters[1,4,5,6], Wolfgang Rathmann[7,8], Harald Grallert[4,5,9], Anna Artati[10], Cornelia Prehn[10], Jerzy Adamski[11,12,13], Christa Meisinger[2*], Jakob Linseisen[1,2*]

*shared last authorship

#Corresponding author, mariana.ponce-de-leon@outlook.com

[1] Institute for Medical Informatics, Biometry and Epidemiology, Ludwig-Maximilians-Universität München, Munich, Germany

[2] Epidemiology, Medical Faculty, Augsburg Universität, Augsburg, Germany

[3] Institute of Translational Genomics, Helmholtz Munich, Munich-Neuherberg, Germany

[4] German Center for Diabetes Research (DZD), Partner Neuherberg, Munich-Neuherberg, Germany

[5] Institute of Epidemiology, Helmholtz Munich, Munich-Neuherberg, Germany

[6]Munich Heart Alliance, German Center for Cardiovascular Health (DZHK E.V., Partner-Site Munich), Munich, Germany

[7]German Diabetes Center (DDZ), Leibniz Center for Diabetes Research, Heinrich Heine University Düsseldorf, Düsseldorf, Germany

[8]German Center for Diabetes Research (DZD), Partner Düsseldorf, Munich-Neuherberg, Germany

[9]Research Unit of Molecular Epidemiology, Helmholtz Munich, Munich-Neuherberg, Germany

[10]Metabolomics and Proteomics Core, Helmholtz Munich, Munich-Neuherberg, Germany

[11]Institute of Experimental Genetics, Helmholtz Munich, Munich-Neuherberg, Germany

[12]Department of Biochemistry, Yong Loo Lin School of Medicine, National University of Singapore, Singapore, Singapore

[13]Institute of Biochemistry, Faculty of Medicine, University of Ljubljana, Ljubljana, Slovenia

Stool sample preprocessing and metabolite measurement

Stool sample processing for metabolomics analysis in the KORA FF4 study was described before in Mitry et al.[1] and is presented here verbatim.

Frozen human stool samples in a weight range of 136 to 143 mg were weighed and were placed into 2 ml homogenization tubes containing ceramic beads with a diameter of 1.4 mm (Precellys Ceramic Kit 1.4 mm, 50x 2,0 ml tubes, Peqlab). Water with a ratio of 12.5µl/mg stool was added into the tubes. The samples were then homogenized in Precellys 24 homogenizer (PEQLAB Biotechnology GmbH, Germany) equipped with an integrated cooling unit for 3 times 20 s at 6,500 rpm, with 15 s intervals between the homogenization steps. After homogenization, 450 µl stool homogenate were transferred into 0.5 ml Eppendorf tube for stool dry mass determination, and 100 µl of the homogenate were pipetted onto a 2 ml 96- deep well plate for non-targeted metabolomics analysis.

In addition to samples from this study, a human reference plasma sample (Seralab, West Sussex, UK) and another reference of human stool (Seralab, West Sussex, UK) were pipetted into 1 and 6 wells of the 96- deep well plate, and were extracted as samples of the study. These samples served as technical replicates throughout the data set to assess process variability. LC-MS/MS based techniques are liable, to a greater or lesser extent, to degradation of instrument performance over time, e.g., columns become contaminated, the response of MS can decline over time for similar reason. Aliquots of a reference plasma sample were included across batches of different studies running on our non-targeted metabolomics platform, upon various matrices of sample of the studies. These samples were examined against a set of predefined criteria, including retention time, mass accuracy, fragmentation pattern, and resolution of some selected compounds in the reference samples. In addition to those samples, 100 µL of water was extracted the same way and placed in 6 wells of 96-well plate to serve as process blanks.

Protein was precipitated and the metabolites in the stool homogenates were extracted with 475 µL methanol, containing 4 recovery standards to monitor the extraction efficiency. The extraction efficiency was monitored by assessing the performance of 4 standard compounds which spanned wider range of metabolite classes. The assessment parameters include chromatogram peak shapes, area under the curves of the chromatograms, retention times, mass accuracies, and fragmentation pattern of the compounds. After centrifugation, the supernatant was split into 4 aliquots of 100 µL each onto two 96-well microplates. Two for analysis by 2 separate reverse phase (RP)/UPLC-MS/MS methods with positive ion mode electrospray ionization (ESI), 1 for analysis by (RP)/UPLC-MS/MS with negative ion mode ESI, and 1 for analysis by (HILIC)/UPLC-MS/MS with negative ion mode ESI. Sample extracts were dried on a TurboVap 96 (Zymark, Sotax, Lörrach, Germany). To minimize human error, liquid handling was performed on an automated MicroLab STAR® robot (Hamilton Bonaduz AG, Bonaduz, Switzerland).

All analytical methods utilized a Waters ACQUITY ultra-performance liquid chromatography (UPLC) and a Thermo Scientific Q-Exactive high resolution/accurate mass spectrometer interfaced with a heated

electrospray ionization (HESI-II) source and Orbitrap mass analyzer operated at 35,000 mass resolution. Prior to the UPLC-MS/MS runs, the dried extract samples were reconstituted with 80 µL of solvents compatible with each of the 4 methods Each reconstitution solvent contained a series of labeled standard compounds at fixed concentrations to monitor the performance of the metabolomics analysis. One aliquot was analyzed using acidic positive ion conditions, chromatographically optimized for more hydrophilic compounds. In this method, the extract was gradient eluted from a C18 column (Waters UPLC BEH C18-2.1 x 100 mm, 1.7 µm) using water and methanol, containing 0.05% perfluoropentanoic acid (PFPA) and 0.1% formic acid (FA). Another aliquot was also analyzed using acidic positive ion conditions; however, it was chromatographically optimized for more hydrophobic compounds. In this method, the extract was gradient eluted from the same afore mentioned C18 column using methanol, acetonitrile, water, 0.05% PFPA and 0.01% FA and was operated at an overall higher organic content. Another aliquot was analyzed using basic negative ion optimized conditions using a separate, dedicated C18 column. The basic extracts were gradient eluted from the column using methanol and water, but with 6.5 mM Ammonium Bicarbonate at a pH of 8. The fourth aliquot was analyzed via negative ionization following elution from a HILIC column (Waters UPLC BEH Amide 2.1x150 mm, 1.7 µm) using a gradient consisting of water and acetonitrile with 10 mM ammonium formate, pH 10.8. The MS analysis alternated between MS and data-dependent MSn scans using dynamic exclusion. The scan range varied slightly between methods but covered 70-1000 m/z.

Raw data was extracted, peak-identified and QC processed using Metabolon's hardware and software (Metabolon, Inc., North Carolina, USA). Compounds were identified by comparison to library entries of purified standards or recurrent unknown entities based on 3 criteria: retention index within a narrow RI window of the proposed identification, accurate mass match to the library +/- 10 ppm, and the MS/MS forward and reverse scores between the experimental data and authentic standards. The MS/MS scores are based on a comparison of the ions present in the experimental spectrum to the ions present in the library spectrum. While there may be similarities between these molecules based on one of these factors, the use of all three data points can be utilized to distinguish and differentiate biochemicals.

MOFA (Multi-Omics Factor Analysis) Model

MOFA model was created following the tutorial "Training a MOFA model in R" (https://biofam.github.io/MOFA2/tutorials.html). Data, model and train data options were set to default. Model characteristics: Number of views: 2; Views names: Blood metabolomics Stool metabolomics; Number of features (per view): 146, 376; Number of groups: 1; Number of samples: 1,370; Number of factors: 20.

Supplementary figures

Figure S1. Study sample (left) and upset plot of MetS components and their combinations in participants classified as having MetS (right)

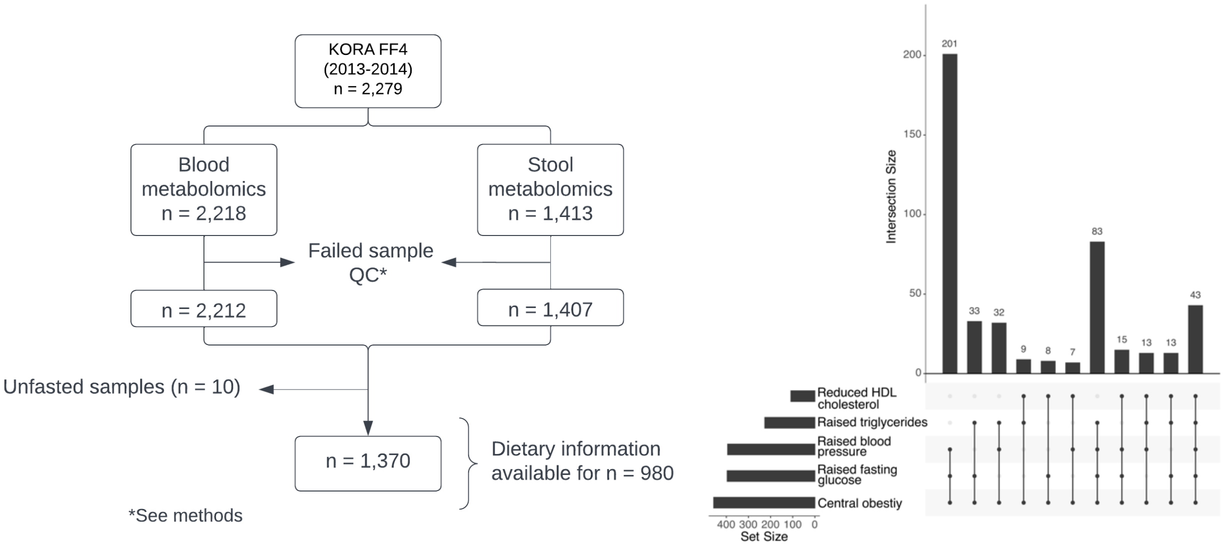

Figure S2. Missing values in stool metabolomics data

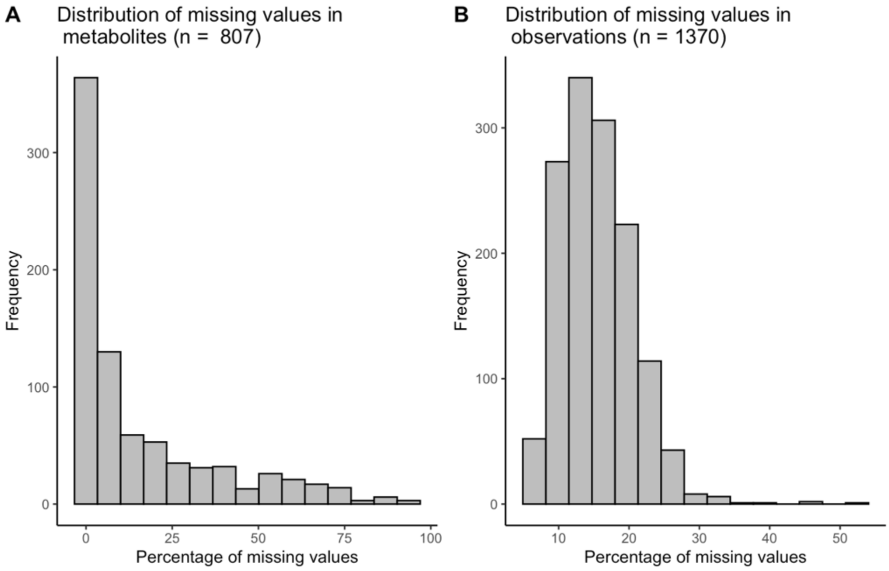

Figure S3. Imputation strategy for missing values in stool metabolomics

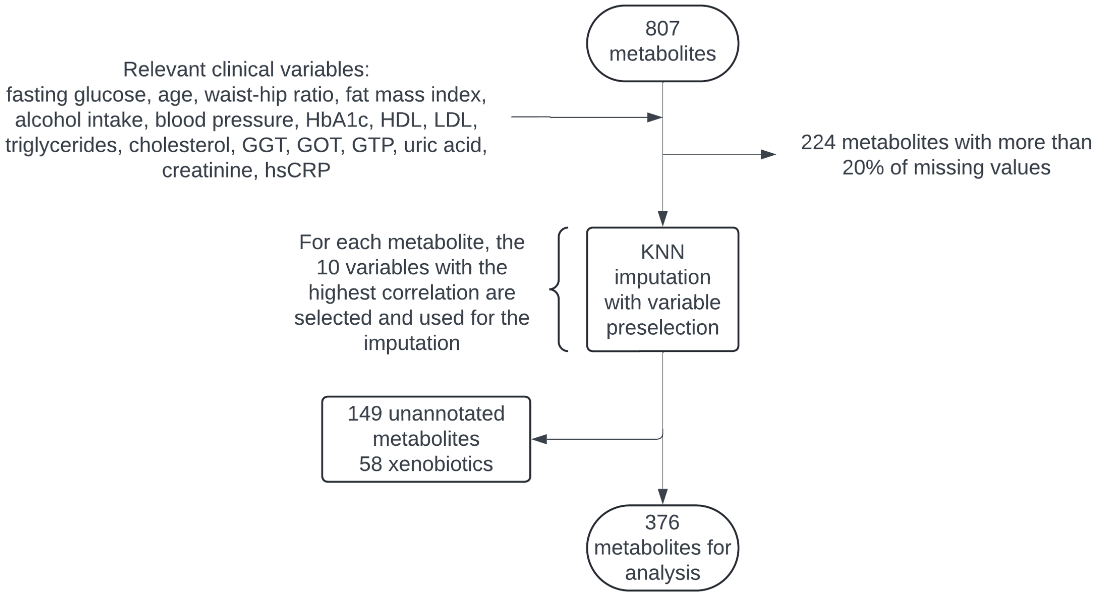

Figure S4. QQ Plot of Mahalanobis distances in blood metabolomics for extreme outlier identification

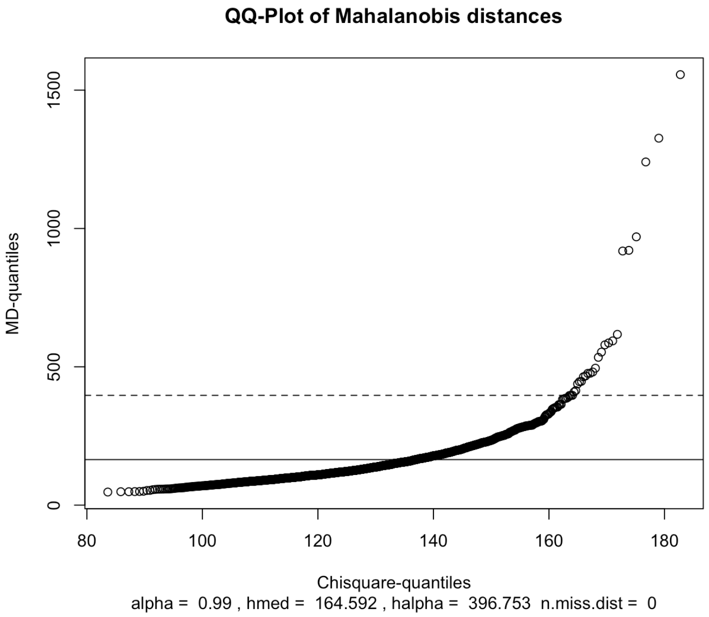

Figure S5. Partial correlation between stool and blood metabolites

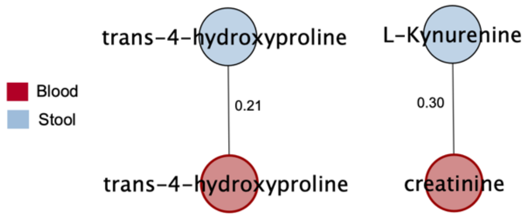

Figure S6. Variance explained by each factor in each data modality (blood/stool) in the MOFA model

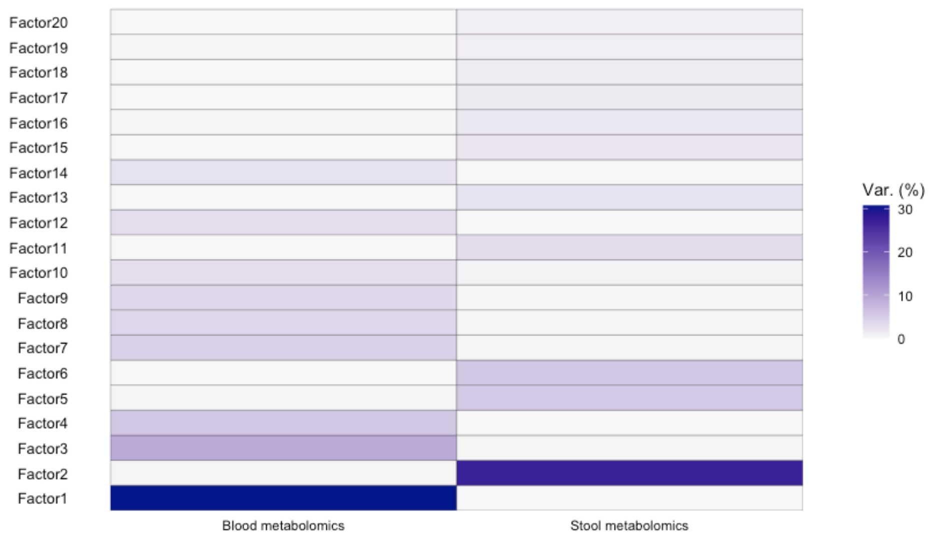

Supplement: Supplementary file 1 — Supplementary Material 1 [file 11306_2024_2166_MOESM1_ESM.pdf]
